# *Mycobacterium bovis* BCG osteoarticular infection complicating immune therapy for bladder cancer: a case report

**DOI:** 10.5194/jbji-6-107-2021

**Published:** 2021-02-22

**Authors:** Rebecca Stern, Clay Roscoe, Elizabeth A. Misch

**Affiliations:** 1 Department of Medicine, University of Wisconsin School of Medicine and Public Health, Madison, Wisconsin, United States; 2 Family Medicine Residency of Idaho, Boise, Idaho, United States

## Abstract

Osteoarticular infection with *Mycobacterium bovis* (*M. bovis*) is a rare complication of bladder cancer treatment with intravesical Bacillus Calmette–Guèrin (BCG). We describe a case of disseminated *Mycobacterium bovis* BCG infection masquerading as a chronic prosthetic joint infection in a patient with several risk factors for progressive mycobacterial infection.

22 February 2021

## 1 Introduction

Bacillus Calmette–Guèrin (BCG), a live, attenuated strain of *Mycobacterium bovis* (*M. bovis*), is recommended as therapy for non-muscle-invasive bladder cancer (Lamm, 2000). Intravesical BCG generates a robust inflammatory response associated with destruction of urothelial cancer cells (Liu et al., 2019). Common complications of BCG immune therapy include malaise and cystitis (Liu et al., 2019). Rare complications include bladder ulceration, sepsis, and dissemination of BCG to distant sites where infectious foci may persist for months or years before symptoms develop (Liu et al., 2019; Pérez-Jacoiste Asín et al., 2014). The long time interval between infection and clinical illness may mislead clinicians unaware of remote BCG exposure.

## 2 Case presentation

**Figure 1:**
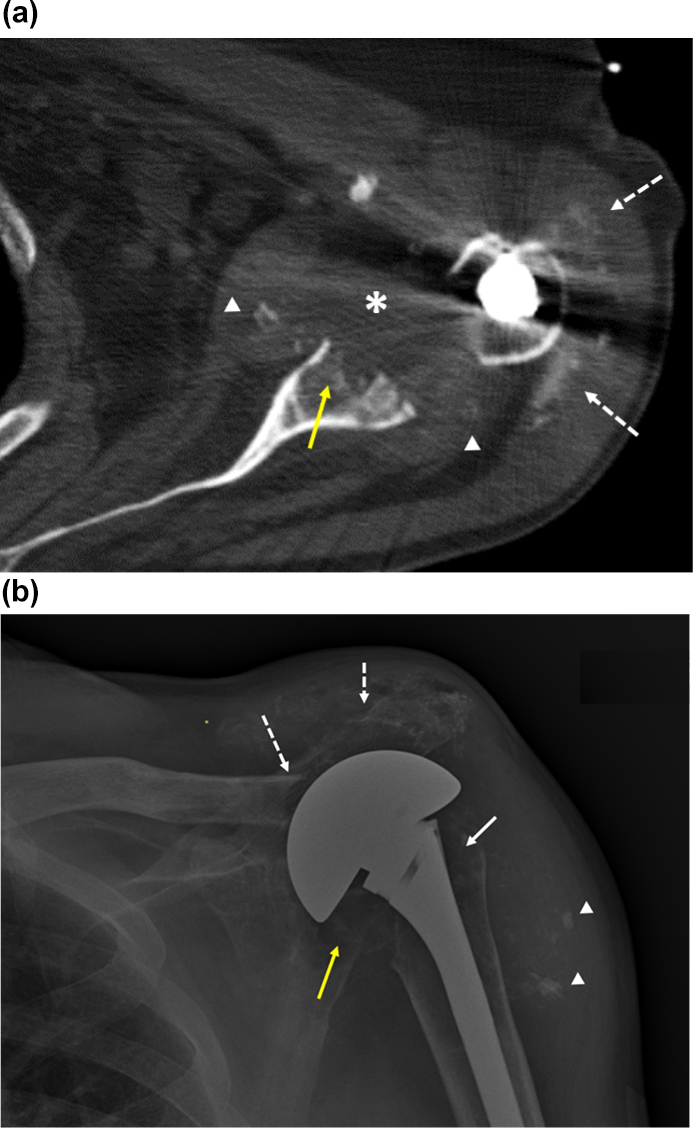
Imaging of the left shoulder before arthroplasty removal. **(a)** Axial CT image of the left shoulder demonstrating erosion of the anterior and central glenoid (solid yellow arrow) with heterogeneously hypoattenuating material centered within the glenohumeral joint space, suggestive of complex effusion (*) and multiple intra-articular fragments of ossific debris (arrowheads) abutting the margins of the thickened joint capsule. This appearance would be most suspicious for septic arthritis, though neoplasm with necrosis could have a similar appearance. Apparent linear and punctate calcification within the subacromial–subdeltoid bursa (dashed white arrows) would be most compatible with displaced articular debris and heterotopic soft tissue ossification, chondroid neoplasm not excluded. Streak artifact from the arthroplasty humeral component limits assessment of fine detail. **(b)** Anteroposterior radiograph of the left shoulder demonstrating erosive changes and extensive demineralization of the left proximal humerus (solid white arrow) with left shoulder arthroplasty hardware in place. Osseous fragmentation and erosion at the acromioclavicular joint, with secondary acromioclavicular joint separation (dashed white arrows), and ossific debris in the subacromial–subdeltoid bursa and/or soft tissue heterotopic ossification (arrowheads). There is additionally erosion and fragmentation of the glenoid (yellow arrow).

An 86-year-old male presented to the hospital with hypotension, chronic left shoulder pain, and an associated effusion in 2014. Remotely, he had undergone left shoulder acromioplasty, followed by hemiarthroplasty in 2010, for severe rotator cuff arthropathy. In 2012, he developed pain at the site of the left shoulder after a fall. Shoulder radiographs showed a stable hemiarthroplasty without evidence of complication. The shoulder pain persisted, and he gradually developed swelling over the left shoulder joint. In 2013, computed tomography (CT) of the shoulder showed a lytic destructive process involving the scapula associated with a large extra-articular proliferative soft tissue process centered on the glenohumeral joint. The mass measured 12×12×6 cm and contained internal calcifications (Fig. 1a). Lysis and fragmentation of adjacent bony structures (especially the acromion) were noted. A neoplastic process such as chondrosarcoma was suspected. A subsequent CT-guided biopsy of the left clavicle lytic lesion was nondiagnostic.

Four months prior to admission, the patient underwent a second, open biopsy of the left shoulder mass and distal clavicle. Bacterial cultures were negative. No fungal or mycobacterial cultures were obtained. Pathology from the clavicle demonstrated multiple non-necrotizing granulomata. Special stains on the formalin-fixed tissues were negative for fungal and mycobacteria organisms.

The past medical history included diabetes complicated by retinopathy, atrial fibrillation, diastolic heart failure, stage III chronic kidney disease, chronic obstructive pulmonary disease, penicillin allergy, and previously treated bladder cancer.

On examination, a draining sinus tract over the left deltoid was noted. The tract had appeared after the open biopsy 4 months previously. A radiograph of the left shoulder demonstrated erosive changes and extensive demineralization of the left proximal humerus with left shoulder arthroplasty hardware in place (Fig. 1b), erosion and separation of the acromioclavicular joint, ossific debris in the subacromial–subdeltoid bursa, and destruction of the glenoid. The findings were felt to be consistent with hardware-associated osteomyelitis. Inflammatory markers were elevated (C-reactive protein 19.5 mg/dL; erythrocyte sedimentation rate 46 mm/h). Bacterial culture of sinus tract fluid yielded *Proteus mirabilis*.

The patient was taken to the OR for hemiarthroplasty removal, biopsy of the distal clavicle, and placement of an antibiotic-impregnated cement spacer. Four days later, he underwent a second irrigation and drainage, at which time the spacer was removed. Pathology on the resected bone revealed granulomas. Operative samples grew *Peptostreptococcus*. Fungal cultures were negative. No mycobacterial cultures were obtained. He received intravenous clindamycin and aztreonam for approximately 6 weeks, with minimal change in symptoms or inflammatory markers. Subsequently, he was treated with vacuum-assisted wound closure and required several additional washouts for bleeding into the shoulder joint space, along with intermittent courses of oral and IV antibiotic therapy.

**Figure 2:**
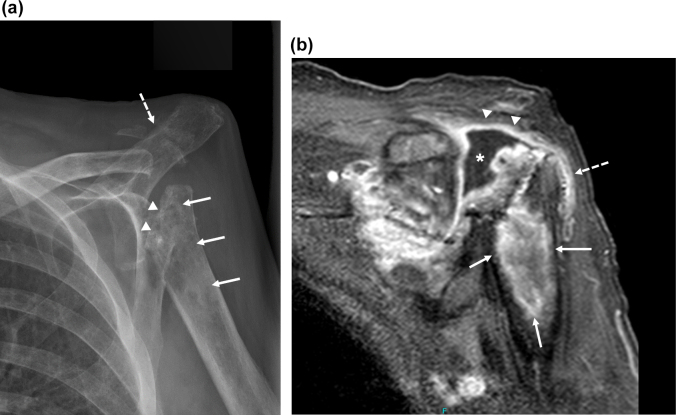
Imaging of the left shoulder 18 months after arthroplasty removal. **(a)** Anteroposterior radiograph of the left shoulder demonstrating erosion of the left proximal humerus at the site of explanted arthroplasty hardware (arrowheads) with extensive osseous mottling, demineralization, patchy sclerosis, and cortical thinning of the proximal humeral metadiaphysis (white arrows). Osseous fragmentation and erosion at the acromioclavicular joint, with secondary AC joint separation (dashed white arrow). These findings are highly suspicious for septic arthritis with osteomyelitis. **(b)** Coronal T1-weighted magnetic resonance image of the shoulder with fat saturation demonstrating peripheral gadolinium contrast enhancement (arrowheads) surrounding a moderate glenohumeral joint effusion in the location of the eroded humeral head (*) with extension to the subacromial–subdeltoid bursa (dashed arrow) and in continuity with a large, rim-enhancing proximal humeral metadiaphyseal intra-osseous abscess (white arrows).

Eighteen months after arthroplasty removal, the patient was readmitted to the hospital with persistent left shoulder pain and effusion, 38 kg weight loss, and malaise. A radiograph of the left shoulder revealed erosion of the left proximal humerus at the site of explanted arthroplasty hardware, along with other findings highly suspicious for osteomyelitis and septic arthritis of the proximal humerus and acromioclavicular joint (Fig. 2a). Magnetic resonance imaging (MRI) demonstrated a peripherally enhancing glenohumeral joint effusion and intra-humeral abscess (Fig. 2b). An aspirate of synovial fluid containing 7000 white cells/mm${}^{3}$ (76 % neutrophils) was pan-cultured. *Cutibacterium acnes* and a *Kocuria* species were recovered from bacterial culture. After 18 d, growth from the mycobacterial cultures of the shoulder joint aspirate was observed; the organism was identified as the *Mycobacterium tuberculosis* (*MTb*) complex by a commercial DNA probe assay. The patient's QuantiFERON^®^-TB Gold blood test was negative. Chest imaging showed no evidence of tuberculosis in the lungs, pleura, or lymph nodes. Sputum samples were submitted for acid fast stain and culture, both of which were negative. Abdominal CT imaging revealed cirrhosis and ascites, previously undiagnosed.

Resistance testing to all first-line anti-mycobacterial drugs was requested, and preliminary results were reported 27 d after cultures became positive (final results were available 42 d after culture positivity). The isolate was susceptible to isoniazid, rifampin, and ethambutol but resistant to pyrazinamide. These results raised suspicion for infection due to *M. bovis*, a member of the *MTb* complex, which is intrinsically resistant to pyrazinamide. Using spacer oligonucleotide typing (spoligotyping) and analysis of mycobacterial interspersed repetitive units (MIRU), the isolate was subsequently confirmed to be the BCG strain of *M. bovis*.

Detailed review of the patient's medical history revealed that he had been treated for urothelial carcinoma with transurethral bladder resection and BCG therapy in 1996. In 2012, the bladder cancer had recurred and was treated with a second course of BCG, which was complicated by persistent cystitis and bladder ulcerations.

A liver-sparing anti-tuberculosis regimen (rifampin, ethambutol, and levofloxacin) was initiated, which the patient tolerated well. However, because of his symptoms of cachexia and weakness, he elected to enter hospice care. All anti-mycobacterial drugs were discontinued, and he expired 13 d after hospital discharge.

## 3 Discussion

This report describes a patient with diabetes, renal failure, and unrecognized cirrhosis who presented with osteoarticular infection 2 years after *M. bovis* BCG exposure. Infection of prosthetic joints is a very rare complication of BCG therapy (Williams et al., 2019; Nguyen et al., 2019). The host features associated with an increased likelihood of BCG infection after bladder cancer immunotherapy remain unclear (Hogan et al., 2017; Pérez-Jacoiste Asín et al., 2014), perhaps because of the relative infrequency of this disease. In contrast, risk factors for progressive disease due to *M. tuberculosis* are well characterized and include diabetes, malnutrition, renal failure, cirrhosis, advanced age, HIV, and the use of tumor necrosis factor (TNF) inhibitors and corticosteroids (Hogan et al., 2017). In the absence of formally demonstrated risk factors for progressive BCG infection, we suggest tuberculosis risk factors may serve as reasonable proxies.

As this case illustrates, mycobacterial joint infections are typically indolent, and patients may present with chronic effusions, draining sinus tracts, regional lymphadenopathy, and even occasional B symptoms. Our patient experienced weight loss of 38 kg over the course of his illness. Laboratory evidence of inflammation, such as elevated synovial fluid or peripheral white cell counts, is often less striking than that seen in typical bacterial joint sepsis (Hogan et al., 2017). In patients infected with BCG, the tuberculin skin test (also known as the purified protein derivative, or PPD, test) may be positive, but *M. tuberculosis*-specific interferon-gamma release assays (IGRAs) are typically negative. In contrast, both tests are usually positive in tuberculosis-infected patients.

The laboratory diagnosis of mycobacterial infection, both at the genus and species level, remains imperfect. The finding of granulomas on pathology is quite sensitive (86 %), but not specific (Pérez-Jacoiste Asín et al., 2014). *M. bovis* is a member of the *M. tuberculosis* complex and the different species within this group cannot be distinguished using currently available commercial probe assays. The sensitivity of either polymerase chain reaction (PCR) or culture for BCG identification is approximately 40 % (Pérez-Jacoiste Asín et al., 2014). On culture, *M. bovis* and BCG are classically resistant to pyrazinamide. The BCG strain can be distinguished from *M. bovis* using genotyping methods, such as spoligotyping and MIRU analysis (Hlavsa et al., 2008).

Failure to consider mycobacteria in the etiology of osteoarticular infection and limitations in laboratory techniques to identify mycobacteria have long been challenges to diagnosis. Fortunately, molecular techniques for mycobacterial identification are now widely available and may supplement older, culture-based methods. In the patient described in this report, technical shortcomings were not at issue. Instead, the diagnosis of mycobacterial infection was not initially considered, and relapsing episodes of infection were ascribed to a variety of bacteria over time. In retrospect, these organisms were mere contaminants and distracted from the true diagnosis. The finding of granulomas, a pathologic hallmark of mycobacterial disease, on bone biopsy should have prompted earlier consideration of mycobacterial infection in a patient with prior BCG exposure. Other overlooked clues included progressive infection despite antibiotic treatment and the presence of multiple risk factors for mycobacterial disease, including cirrhosis, renal failure, and diabetes.

## 4 Conclusion

Clinicians should maintain awareness of the risk factors and clinical features of mycobacterial infection and seek to elicit a history of exposure, particularly in patients not responding to routine anti-bacterial agents or with characteristic granulomatous histopathology on tissue biopsy.

## Data Availability

No data sets were used in this article.
